# Retraction: The Interplays between Autophagy and Apoptosis Induced by Enterovirus 71

**DOI:** 10.1371/journal.pone.0246985

**Published:** 2021-02-09

**Authors:** The PLOS ONE Editors

Following the publication of this article [1], concerns were raised regarding results reported in Figs 1, 2, 3, 5, 6, and 7. Specifically,

- In Fig 1C, the background directly surrounding the bands presented in the LC3I / LC3II blot does not appear to match the overall background of the blot.
- In Fig 2B, multiple repetitive clusters of signal have been detected within and between the reported FACS data. The similarity of the signal between these panels is greater than would be expected from independent FACS samples.
- The following bands appear similar:
  ○ Fig 3A, P62 panel: lane 3 and lane 7.
  ○ Fig 3C, PARP panel: lane 1 and lane 4 when flipped.
  ○ Fig 3C, Beclin1 panel: lane 1 and lane 3.
  ○ Fig 5A, PARP panel: lane 4 and lane 6.
  ○ Fig 5A, Caspase-p panel: lane 1 and lane 3.
  ○ Fig 5A, LC3-I panel: lanes 1, 2 and lanes 3, 4 respectively.
  ○ Fig 5A, LC3-II panel: lanes 1, 2 and lanes 3, 4 respectively.
  ○ Fig 5A, P62 panel: lanes 5, 6 and lanes 7, 8 respectively.
- The PARP panel of Fig 3A appears similar to the PARP panel of Fig 6A despite being used to represent different experimental conditions.
- The Actin panel of Fig 5A appears similar to the Actin panel of Fig 7A despite being used to represent different experimental conditions.

The original data underlying the published figures are no longer available. The authors provided repeat experiment data to support some of the experiments for which questions were raised, but the data did not resolve the concerns.

In light of the concerns affecting multiple figure panels that question the integrity of these data and the unavailability of the data underlying the published results, the *PLOS ONE* Editors retract this article.

XX, XZ, BW, TW, JiW, HH, JiaW, QJ, and ZZ either did not respond to the retraction or could not be reached.
